# Efficacy and Safety of Low Frequency Whole-Body Electromyostimulation (WB-EMS) to Improve Health-Related Outcomes in Non-athletic Adults. A Systematic Review

**DOI:** 10.3389/fphys.2018.00573

**Published:** 2018-05-23

**Authors:** Wolfgang Kemmler, Anja Weissenfels, Sebastian Willert, Mahdieh Shojaa, Simon von Stengel, Andre Filipovic, Heinz Kleinöder, Joshua Berger, Michael Fröhlich

**Affiliations:** ^1^Institute of Medical Physics, University of Erlangen-Nürnberg, Erlangen, Germany; ^2^Round-Table Whole-Body Electromyostimulation, Erlangen, Germany; ^3^Department of Training Sciences and Sports Informatics, German Sport University Cologne, Cologne, Germany; ^4^Department of Sports Science, University of Kaiserslautern, Kaiserslautern, Germany

**Keywords:** whole-body electromyostimulation, EMS, muscle mass, fat mass, low back pain, cardio-metabolic, risk factors, creatine kinase

## Abstract

Exercise positively affects most risk factors, diseases and disabling conditions of middle to advanced age, however the majority of middle-aged to older people fall short of the exercise doses recommended for positively affecting cardio-metabolic, musculoskeletal and neurophysiological fitness or disabling conditions. Whole-Body Electromyostimulation (WB-EMS) may be a promising exercise technology for people unable or unmotivated to exercise conventionally. However, until recently there has been a dearth of evidence with respect to WB-EMS-induced effects on health-related outcomes. The aim of this systematic review is to summarize the effects, limitations and risks of WB-EMS as a preventive or therapeutic tool for non-athletic adults. Electronic searches in PubMed, Scopus, Web of Science, PsycINFO, Cochrane and Eric were run to identify randomized controlled trials, non-randomized controlled trials, meta-analyses of individual patient data and peer reviewed scientific theses that examined (1) WB-EMS-induced changes of musculoskeletal risk factors and diseases (2) WB-EMS-induced changes of functional capacity and physical fitness (3) WB-EMS-induced changes of cardio-metabolic risk factors and diseases (4) Risk factors of WB-EMS application and adverse effects during WB-EMS interventions. Two researchers independently reviewed articles for eligibility and methodological quality. Twenty-three eligible research articles generated by fourteen research projects were finally included. In summary, thirteen projects were WB-EMS trials and one study was a meta-analysis of individual patient data. WB-EMS significantly improves muscle mass and function while reducing fat mass and low back pain. Although there is some evidence of a positive effect of WB-EMS on cardio-metabolic risk factors, this aspect requires further detailed study. Properly applied and supervised, WB-EMS appears to be a safe training technology. In summary, WB-EMS represents a safe and reasonable option for cohorts unable or unwilling to join conventional exercise programs. However, much like all other types of exercise, WB-EMS does not affect every aspect of physical performance and health.

## Introduction

The growing prevalence and severity of chronic diseases along with the multi-morbidity of our adult population are creating an ever-greater strain on the healthcare systems of western nations. With respect to the German population, more than two thirds of the population aged 50 years suffer from two and more diseases with about 20–25% of them suffering from five and more diseases (Robert-Koch-Institut, 2014). Physical activity and exercise is undisputedly the most comprehensive therapeutic agent for an overaged and fundamentally sedentary society. Indeed, reviewing the present literature (Pedersen and Saltin, 2006; Börjesson et al., 2010), there is a consensus that exercise positively affects most, if not all, risk factors, diseases and disabling conditions of middle to advanced age. Addressing all favorable effects of exercise in detail would go far beyond the scope of this review. However, the finding that different outcomes ranging from various musculoskeletal (e.g., Marques et al., 2011; Peterson et al., 2011; Searle et al., 2015), cardiometabolic (e.g., ExtraMatch-Collaborative, 2006; Ismail et al., 2012; Pattyn et al., 2013; Yang et al., 2014; Inder et al., 2016) physical performance (e.g., Macaluso and De Vito, 2004; Straight et al., 2016; Sherrington et al., 2017), and cognitive function (e.g., Chieffi et al., 2017a,b) parameters can be positively affected by physical activity/exercise, might demonstrate the enormous comprehensive potential of exercise on health-related parameters.

Unfortunately, even the majority of prime-agers in Germany or the USA (Carlson et al., 2010; Statistisches-Bundesamt, 2016) fall short of the exercise doses recommended for positively impacting cardio-metabolic, musculoskeletal and neurophysiological fitness or disabling conditions (AHHS, 2008; Chodzko-Zajko et al., 2009; Garber et al., 2011). Moreover, a considerable proportion of people at advanced age (Carlson et al., 2010; Statistisches-Bundesamt, 2016), i.e., individuals with a more pronounced need for health-enhancing approaches, might be unable or unmotivated to perform conventional exercise programs with the necessary dose. Frequent reasons given for abstaining from exercising are time constraints, physical limitations, risk of injuries and/or little enthusiasm for exercise conducted alone (Rütten et al., 2009; Rodrigues et al., 2017). Hence, innovative, time-efficient, joint-friendly, highly individualized and closely supervised exercise technologies might be a good choice for people looking to maintain or improve their health and physical fitness. Whole-Body Electromyostimulation (WB-EMS), a technology that is based on the recognized local EMS but addresses up to nine (main) muscle groups simultaneously each with dedicated intensity might be such a choice. Indeed, considering the rather low exercise volume of 1–2 sessions of 20 min/week, the low voluntary intensity and the high degree of supervision and individualization of present WB-EMS settings might attract people with low affinity to conventional exercise programs.

However, until recently there has been a considerable lack of evidence with respect to WB-EMS-induced effects on health-related outcomes. The aim of this systematic review is therefore to provide evidence for the effects, limitations and risks of WB-EMS as a preventive or therapeutic tool for non-athletic adults. So as to present reliable and representative data, we focus on WB-EMS frequencies 80–85 Hz, (considered as “low-frequency WB-EMS), the predominantly applied type of WB-EMS in commercial settings and—to our best knowledge—the only type of WB-EMS that has been evaluated in scientific studies.

In order to present a comprehensible overview, we have structured this systematic review in four sections. Each of these sections are reported and discussed separately:

1. WB-EMS-induced changes of musculoskeletal risk factors and diseases.
2. WB-EMS-induced changes of functional capacity and physical fitness.
3. WB-EMS-induced changes of cardio-metabolic risk factors and diseases.
4. Risk factors of WB-EMS application and adverse effects during WB-EMS interventions.

## Methods

This systematic review follows the Preferred Reporting Items for Systematic reviews and Meta-Analyses guidelines (PRISMA) (Moher et al., 2009). Of importance, we conducted a joint research strategy for topics (1) – (3) and for the aspect of adverse effects during WB-EMS interventions. “Risk factors of WB-EMS applications” were addressed by a separate literature research.

### Literature search strategy

A comprehensive search of electronic databases was conducted through PubMed, Scopus, Web of Science, PsycINFO, Cochrane and Eric for all articles published in English and German up to October 30, 2017, on the effect of WB-EMS on muscle mass, physical functioning, bone mass, cardio-metabolic diseases and low back pain. The literature search was constructed around search terms for “Whole-Body Electromyostimulation,” “muscle mass,” “muscle strength/physical functioning,” “bone mineral density,” “cardio-metabolic diseases,” “low back pain” consistently with the focus on “adults.” A standard protocol for this search was developed and controlled vocabulary (Mesh term for MEDLINE) was used. Key words and their synonymous were used to sensitize the search by using the following query: (“Whole-Body Electromyostimulation” or “whole body electrostimulation” or “whole body myostimulation” or “extended Electromyostimulation”) AND (“muscle mass” or “lean body mass” or “musculoskeletal” or “skeleton” or “strength” or “physical capacity” or “physical function” or “bone mineral density” or “bone mass” or “osteoporosis” or “sarcopenia” or “back pain” or “dorsal pain” or “cardio-metabolic” or “metabolic” or “cardiac” or “heart diseases” or “obesity” or “fat” or “diabetes” or “metabolic syndrome” or “waist circumference” or “blood pressure” or “cholesterol” or “triglycerides” or “insulin” or “inflammation”), AND (“human” or “adults”). Subsequently, a German translation of the search terms was used to search relevant databases in German. Additionally, reference lists of the included studies were searched manually. Duplicate publications were identified by comparing author names, interventions comparisons, outcome measures, publication dates, sample sizes, and outcomes.

**Figure 1:**
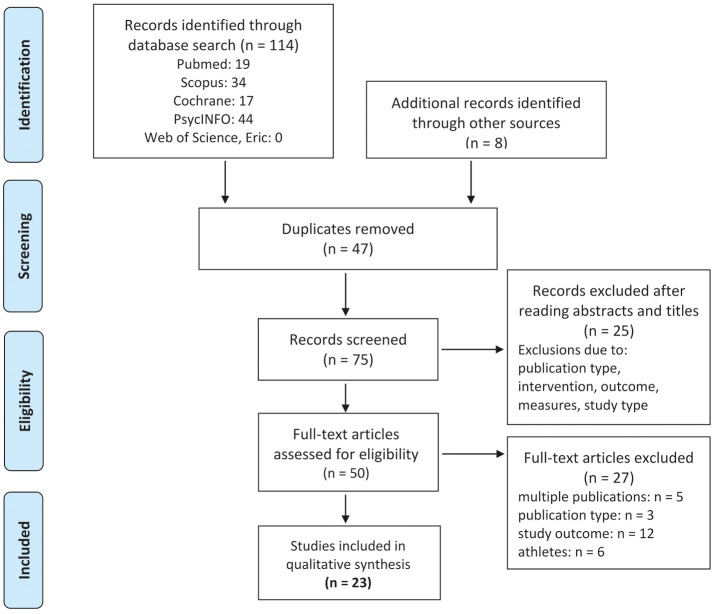
Flow diagram of search process according to PRISMA (Moher et al., 2010).

Randomized controlled trials (RCT), non-randomized controlled trials (NCT), meta-analysis of individual patient data and peer reviewed scientific theses that examined the effect of WB-EMS on the above listed parameters among human adults were included in the review. When there were multiple publications from a single project, the largest study was included. When articles generated by one projects focus on different study endpoints that were addressed in this review, all the articles were included. Review articles, observational studies, case reports, editorials, conference abstracts, animal studies and letters were excluded. Cohorts with a non-athletic lifestyle were included. Athletes, sport students or physical education students were not included (Figure 1).

### Data extraction

Two independent reviewers (WK, MS) responsible for eligibility screened the titles and abstracts. A specialized extraction form was designed and used to list the methodological details for each study: authors, country and year of publication; details of the study including study design, study objectives, sample size, inclusion and exclusion criteria for participants, participant characteristics (i.e., age, weight, height), description of WB-EMS intervention (i.e., frequency, intensity), number of participants at baseline and study completion (including number of withdrawals), risk assessment, types of outcome variables assessed and their values at baseline and study completion.

### Outcome measures

The outcomes in this report are changes of:

(1)Body composition, including lean body mass (LBM), muscle mass, appendicular skeletal muscle mass (ASMM), total and abdominal fat mass (or rate) and bone mineral density (BMD), (2) Strength and endurance related parameters, physical functioning (e.g., gait speed, SPPB), (3) Unspecific chronic low back pain, and (4) Cardio-metabolic parameters and related factors, from baseline and study end. Further, we address (5) potential risk factors of acute WB-EMS application and adverse effects during WB-EMS interventions.

### Quality assessment

All the articles that satisfied the predefined inclusion criteria (Figure 1) were independently assessed for risk of bias by two independent raters (WK and SvS) using the PEDro scale (Sherrington et al., 2000; de Morton, 2009). This list consists of eleven quality criteria that evaluate different methodological aspects of the studies. Of importance, one of the eleven criteria (i.e., blinding of the trainers) is not applicable for this type of intervention, and so this item was excluded. Difference in the quality assessment between the raters were discussed with a third assessor until a consensus was reached.

### Statistical analysis

Effect sizes (ES) as defined as standardized mean differences (SMD) were calculated using Cohen‘s d (Cohen, 1988). ES ≥ 0.5 were considered as moderate; ES ≥ 0.8 were considered as high. Two projects did not provide standard deviations (SD) (van Buuren et al., 2013, 2014; Özdal and Bostanci, 2016), that are however essential for the calculation of Cohen‘s d (van Buuren et al., 2013, 2014; Özdal and Bostanci, 2016). One of the authors (Özdal and Bostanci, 2016) retrospectively provided the corresponding information upon request. According to recent suggestions (Furukawa et al., 2006), SD of the remaining studies were estimated considering data of studies with the identical topic (Furukawa et al., 2006). Of importance, we imputed the mean SD provided by the available studies in the equation, as suggested by the Cochrane Collaboration (Cochrane, 2016).

## Results

### Characteristics and methodologic quality of the trials

Our search strategy finally identified twenty-three eligible research articles and (masters) theses (Grützmacher, 2002; Vatter, 2003; Fritzsche et al., 2010; Kemmler et al., 2010a,b, 2012a, 2013, 2014, 2015c,d, 2016b,c,d,e, 2017a,b,c; Kemmler and Von Stengel, 2013; van Buuren et al., 2013, 2014; Von Stengel et al., 2015; Özdal and Bostanci, 2016; Wittmann et al., 2016) generated by fourteen research projects numbered and listed in alphabetic order (Table 1).

**Table 1:** Study characteristics.

**Authors**	**Study-Design**	**Sample size (*n*)**	**Status**	**Sex, Age (MV ± SD)**	**Control**	**Intervention**	**PEDro Scale**
Fritzsche et al., 2010	NCT	15/-	Cardiac patients, untrained	M + W, 56 ± 16 years	No CG	80–100 dynamic exercises: 20 min, **bipolar, rectangular, 80 Hz**.; impulse width n.g.; 4 s impulse−4 s rest, intensity: RPE 8 (maximum 12), 2 sessions/week, 6 months	3
Grützmacher, 2002	NCT	49/-	back pain, untrained	47 ± 9	No CG	12 isometric exercises, 45 min **bipolar, rectangular, 350** μ**s 80 Hz**. 4 s impulse−2 s rest, maximum RPE (+15 min of regenerational WB-EMS); 2 sessions/weeks, 6 weeks	3
Kemmler et al., 2010b, 2015c	RCT	15/15	Healthy, moderately trained	M, 66 ± 6 years	Maintain exercise	1–2 sets, 10 exercises, 6–8 reps in a standing position; 10 min **bipolar, rectangular, 350** μ**s**; 10 min with **85 Hz**. 4 s impulse−4 s rest, and 10 min with 7 Hz. continuous impulse; intensity: RPE 6–7 (hard+ to very hard; Borg CR-10), 1.5 sessions/w., 14 w.	8
Kemmler et al., 2010a, 2015c	RCT	14/14	Healthy, untrained	M, 69 ± 3 years.	Semi-active: WBV	10 min crosstrainer (70% VO_2_peak), bipolar, rectangular, 85 Hz, 350 μs; continuous impulse and 15 min 2 sets, 7 exercise, 6–8 reps, **bipolar, rectangular, 85 Hz, 350** μ**s**, 4 s−4 s; intensity: RPE 6–7 (hard+ to very hard, Borg CR-10), 1.5 sessions/w., 14 w.	8
Kemmler et al., 2013, 2014, 2015c; Von Stengel et al., 2015	RCT	38/38	Osteopenia, untrained	W, 75 ± 5 years	Semi-active: wellness	20 min, 1–2 sets of 8–12 exercises with 6–8 reps, **bipolar, rectangular, 85 Hz, 350** μ**s**, 4–6 s impulse−4 s rest; intensity: RPE 6-7 (Borg CR-10), 1.5 sessions/week, 12 months; CG: 2 × 10 weeks with one session/w. slight exercises for well-being	9
Kemmler and Von Stengel, 2013	RCT	23/23	Osteopenia, untrained	W, 75 ± 5 years.	wellness	(see Kemmler et al., 2013, 2014; Von Stengel et al., 2015), however only subject with waist circumferences >80 cm was included	9
Kemmler et al., 2015d, 2016b,c	RCT	23/23	Healthy, untrained	M, 42 ± 6 years	Active: HIT-RT	20 min, 1–2 sets of 12 exercises with 6–8 reps, **bipolar, rectangular, 85 Hz, 350** μ**s**, 6 s impulse−4 s rest; intensity: RPE 7 (very hard, Borg CR-10), 1.5 sessions/week, 14 weeks, CG: “HIT”- RT protocol (2–3 × 30 min/w., 10–13 exercises, 4–12 reps, failure+)	9
Kemmler et al., 2015c	RCT	14/14	Low muscle mass, untrained	W, 76 ± 5 years	Active: WB-EMS	16 min, 1–2 sets of 6–8 reps of 10 low intense movements in a supine position; **bipolar, rectangular, 85 Hz, 350** μ**s**, 4 s−4 s; RPE 6 (hard+, Borg CR-10), 1 session /week, 12 weeks; CG: WB-EMS without any movements in supine position	8
Kemmler et al., 2016e; Wittmann et al., 2016	RCT	25/25	Sarcopenic obesity, untrained	W, 77 ± 4 years.	Inactive	20 min, 1–2 sets of 12 low intense movements with 6–8 reps in a supine position**, bipolar, rectangular, 85 Hz, 350** μ**s**, 4–6 s impulse−4 s rest; intensity: RPE 5–6 (hard-hard+, Borg CR-10), 1 session/week, 26 weeks	9
Kemmler et al., 2017a,c	RCT	33/34	Sarcopenic obesity, untrained	M, 77 ± 5 years.	Inactive	20 min, 1–2 sets of 12 exercises with 6–8 reps, **bipolar, rectangular, 85 Hz, 350** μ**s**, 6 s impulse−4 s rest; intensity: RPE 6–7 (hard+ to very hard, Borg CR-10), 1.5 sessions/week, 16 weeks	9
Kemmler et al., 2017b	Meta-Analysis: 5 studies	23/22	Unspecific chronic low back pain	M+W, 60 years+	Inactive	16–25 min, 1–2 sets of 8–12 low intense movements with 6–8 reps, **bipolar, rectangular, 85 Hz, 350** μ**s**, 4–6 s impulse−4 s rest; intensity: RPE 5–7 (hard- to very hard, Borg CR-10), 1.5 sessions/week, 14–52 weeks	8–9 see above
Özdal and Bostanci, 2016	RCT	20/20	Healthy untrained	W, 33 ± 8	Active: WB-EMS	25 min; 10 min RT with dumbbells, 10 min aerobics, 5 min cooldown, **bipolar, rectangular (?), 80 Hz, impulse width n.g. (standard 350** μ**s?)**, 4 s impulse−4 s rest; intensity: n.g. (adequate?); 3x week, 8 weeks; CG: WB-EMS without exercises	6
van Buuren et al., 2013, 2014	NCT	22/12	Cardiac patients, untrained	M+W, 61 ± 11	Active: local EMS	20 min, **bipolar, 80 Hz, 350** μ**s**, 4 s impulse−4 s rest; intensity: n.g. (self-regulation by participant); passive application without movements? 2 sessions/w.; 10 weeks Local EMS: focus on thigh and gluteus-electrodes	5
Vatter, 2003	NCT	134/10	Healthy, trained	M+W, 43 ± 12	Maintain exercise	12 isometric exercises, 45 min, **bipolar, rectangular, 350** μ**s 85 Hz**. 4 s impulse−4 s rest, maximum RPE; (+15 min of regenerational WB-EMS); 2 sessions/weeks, 6 weeks	4

*NCT, Non-randomized controlled trial; M, men; W, women; n.g., not given, RT, Resistance training; CG, control group*.

There follows a brief outline of the projects summarized in Table 1:

(1) Fritzsche et al. (2010) presented a project that focused on primary prevention in 11 men and 4 women with chronic heart failure (CHF), 27–76 years old. The 6-month study did not set up a control group (CG). Study endpoints focus on endurance parameters and body composition; data for blood pressure and low back pain were also presented.
(2) Grützmacher (Grützmacher, 2002) conducted a 6-week, twice weekly 45 min WB-EMS study that focused on low back pain (LBP) with 31 female and 18 male university employees with LBP, 47 years old on average. The project did not implement a non-training control group. Data presented here refer to low back pain published in a non-published master thesis (Grützmacher, 2002). A short research paper summarizing this work is available online (Boeckh-Behrens et al., 2002).
(3) Kemmler et al. (2010b, 2015c) randomly allocated 30 female participants of the EFOPS exercise study (Kemmler et al., 2015a), 66±6 years old, in a CG that maintained their general training program (2 × 60 min/week) and a group that additionally performed 2 × 20 min WB-EMS for 14 weeks. Primary study endpoints of TEST I (Training and Electromyostimulation Trial I) were body composition and strength development.
(4) The TEST-II project (Kemmler et al., 2010a, 2015c) primarily addressed body composition and—to a lesser degree—strength in untrained men with the Metabolic Syndrome (MetS) according to the International Diabetes Federation (IDF)(Alberti et al., 2006). The project randomly allocated 28 men, 65–75 years old to a WB-EMS and a Whole-Body Vibration (WBV) CG (18 min, 30 Hz) both performing identical slight movements during WB-EMS/WBV application for 14 weeks.
(5) The primary aim of TEST III (Kemmler and Von Stengel, 2013; Kemmler et al., 2013, 2014, 2015c; Von Stengel et al., 2015) was to determine the WB-EMS effects on bone mineral density (BMD) at lumbar spine (LS) and proximal femur in 76 sedentary women 70 years+ with low body mass index (<23 kg/m^2^). Participants were randomly allocated to WB-EMS and CG that conducted similar movements in training blocks with a comparable training volume. Two publications focused on BMD (Kemmler et al., 2013; Von Stengel et al., 2015) and two other contributions (Kemmler and Von Stengel, 2013; Kemmler et al., 2014) reported body composition and strength outcomes. One of the latter studies (Kemmler and Von Stengel, 2013) (6) might be considered as a sub-study of the TEST-III study that included only women with abdominal obesity according to the IDF-criteria (Alberti et al., 2006).
(7) In another project (Kemmler et al., 2015d, 2016b,c) directly compared the effect of 16 weeks of WB-EMS versus high intensity (resistance exercise) training (HIT) “defined as a single set to failure+ protocol” (Steele et al., 2017a). Forty-eight untrained, predominately overweight men, 30–50 years old were randomly assigned to the two study groups that were both very time efficient (i.e., WB-EMS 20 min vs. HIT: 30 min/session). Two studies reported body composition and strength parameters (Kemmler et al., 2015d, 2016c), one study focused on cardio-metabolic outcomes (Kemmler et al., 2016b).
(8) The TEST-V project aimed to determine whether very slight voluntary muscle activation during WB-EMS application increases the WB-EMS effect on muscle function (Kemmler et al., 2015c). Twenty-eight sedentary women 70 years+ with sarcopenic obesity (SO) were randomly assigned to a “passive” WB-EMS group versus an “active” WB-EMS group that performed very slight movements of the upper and lower limbs during the impulse phase, while both groups rested in a supine position on a special chair during the identical WB-EMS application. The primary study endpoint was leg extension strength as determined by an isokinetic leg-press.
(9) The FORMoSA project randomly assigned 75 sedentary women 70 years+ with SO to a (1) WB-EMS (2) WB-EMS and protein supplementation and (3) inactive CG. The 6-month project focused predominately on muscle, fat and functional parameters constituting the sarcopenic obesity definition(s) (Kemmler et al., 2016d,e); however, one article described changes of MetS and related parameters (Wittmann et al., 2016).
(10) The FRANSO project, a 4-month RCT with three study arms aimed to determine the effect of (1) protein supplements (PS) vs (2) WB-EMS and PS vs. (3) a non-training control in 100 untrained men 70+ with SO. Whey protein was supplemented up to a total daily protein intake of 1.7-1.8 g/d/kg body mass. The studies published (Kemmler et al., 2017a,c) focused specifically on body composition and functional parameters constituting the sarcopenic obesity, however, related and alternative body-composition and strength parameters were also reported (Kemmler et al., 2017a).
(11) In order to generate adequate statistical power, the most recently published WB-EMS project (Kemmler et al., 2017b) can be considered as a meta-analysis of individual patient data. The study included participants 60 years+ with moderate-severe unspecific LBP and summarized the results of five projects with five WB-EMS vs. five control groups (Kemmler et al., 2010a,b, 2014, 2016e, 2017c) that listed LBP as an experimental endpoint.
(12) Özdal and Bostanci (2016) applied eight weeks of WB-EMS with 40 sedentary women, 32 ± 8 years old with and without additional, albeit not more closely described, adjuvant moderate intensity RT and step-aerobic exercises. The study focused on total and regional fat reduction, further lean body mass changes were reported.
(13) In two related NCTs van Buuren et al. (2013, 2014) reported the effect of WB-EMS vs. locally applied EMS (thigh and gluteals with the same stimulation parameters) on left ventricular function and peak oxygen consumption in patients with CHF. The twice-weekly WB-/EMS protocol was performed for 10 weeks.
(14) Finally, Vatter (2003) conducted a multicenter NCT with 144 young/middle-aged men and women exercising in commercial fitness facilities. The trial focused on the effect of six weeks of twice-weekly 45 min WB-EMS on various outcomes (i.e., total body fat, maximum strength, back pain, incontinence). Data presented here refer a non-published masters thesis, however a corresponding book (Vatter, 2010) is also available.

In summary, thirteen projects are WB-EMS trials and one study (Kemmler et al., 2017b) is a meta-analysis of individual patient data. Table 1 lists study characteristics and methodological quality according to PEDro scale (Sherrington et al., 2000; de Morton, 2009). Score points vary between four and nine from a maximum of 10 score points feasible for this type of intervention. None of the studies realized blinding of the participants, which is very difficult in this type of intervention studies, however. On the other hand, all the studies gained points for specification of eligibility criteria. Seven studies reported allocation concealment and conducted a blinding of study assessors. Five studies applied the intention to treat principle. Two studies did not implement a control group (Fritzsche et al., 2010; Grützmacher, 2002). Low score points resulted consistently from a lack of randomization and blinding. Nine of the projects generated randomized controlled trials (RCTs), and two projects featured a non-randomized design (Vatter, 2003; van Buuren et al., 2013, 2014). The cohorts included by the WB-EMS trials vary from healthy, untrained middle-aged men (Kemmler et al., 2016c) and/or women (Vatter, 2003; Özdal and Bostanci, 2016), cardiac patients (Fritzsche et al., 2010; van Buuren et al., 2013, 2014), to older untrained cohorts (70 years+) with sarcopenia and osteopenia (Kemmler et al., 2014; Von Stengel et al., 2015) and sarcopenia or sarcopenic obesity (SO) (Kemmler et al., 2016e, 2017c; Wittmann et al., 2016). Correspondingly, study participants' age ranges from 18 to 85 years, with few exceptions (Grützmacher, 2002; Vatter, 2003; Fritzsche et al., 2010; Kemmler et al., 2016c; Özdal and Bostanci, 2016), however, most of the projects focused on participants at least 60 years and older. Although the sample sizes of most studies were only low—moderate, the statistical power to address at least one given study endpoint (e.g. “maximum strength” or “peak oxygen consumption”) was adequate. Of relevance for the corresponding group differences, nine projects implemented “active comparator” CGs. Consequently, study effects, defined as differences between WB-EMS and CG, should be interpreted very cautiously and with careful consideration of the exercise specification of the active CG. With respect to the movements conducted during WB-EMS, apart from one project that did not provide appropriate information (van Buuren et al., 2013, 2014) and two projects by Özdal and Bostanci (2016) and Vatter (2003) that potentially exercised with moderate (voluntary) intensity, all the studies reported that they applied (very) low intensity movements/exercises. WB-EMS impulse specifications were very similar between the projects. Apart from two trials (Grützmacher, 2002; Vatter, 2003) that used a precursor (Body-Transformer, BPL Concept, Köln, Germany), all the other projects used the same low-frequency WB-EMS devices (miha bodytec, Gersthofen, Germany). Further, all the projects conducted supervised sessions with bipolar, predominately rectangular impulse protocols, impulse frequencies of 80 or 85 Hz, and an impulse breadth of 350 μs for 16–45 min. Additionally, all of the projects applied intermitted applications with 4 or 6 s of impulse during the exercise/movement and 2–4 s of rest between the exercises/movements, with three projects (Kemmler et al., 2010a,b; Özdal and Bostanci, 2016) additionally applying continuous WB-EMS with 7 or 85 Hz in the same session. Two trials (Grützmacher, 2002; Vatter, 2003) applied a low intensity cool-down WB-EMS application (100 Hz, bipolar, rectangular, 250 μs, 1 s −1 s) for up to 15 min at the end of sessions. Prescribed training frequency of the projects ranged from one session/week (Kemmler et al., 2016d,e) to three sessions/week (Özdal and Bostanci, 2016), and hence the total WB-EMS volume/week varied from 20 min (Kemmler et al., 2016d,e) to 90–120 min/week (Grützmacher, 2002; Vatter, 2003). Length of the projects differed considerably between 6 weeks (Grützmacher, 2002; Vatter, 2003) and 12 months (Kemmler et al., 2013, 2014, 2015c; Von Stengel et al., 2015) of WB-EMS application. All but one study, which did not comprehensively specify its procedure (Özdal and Bostanci, 2016), used RPE-based approaches to regulate the intensity of their WB-EMS protocol. Of importance, with the exception of studies focusing on CHF-patients (Fritzsche et al., 2010; van Buuren et al., 2013, 2014) and prescribing a lower intensity for their patients, all the projects used perceived exertion specifications of between “hard” (older women with SO, Kemmler et al., 2016e) and “very hard” (untrained men 30–50 years old Kemmler et al., 2016c) or maximum “RPE” (back pain patients and young/middle-aged, fitness trained subjects Grützmacher, 2002; Vatter, 2003).

## Discussion

### (1) WB-EMS-induced changes of musculoskeletal risk factors and diseases

In summary, 11 eligible projects with 19 studies (Grützmacher, 2002; Fritzsche et al., 2010; Kemmler et al., 2010a,b, 2012a, 2013, 2014, 2015c,d, 2016b,c,d,e, 2017a,b,c; Kemmler and Von Stengel, 2013; Von Stengel et al., 2015; Özdal and Bostanci, 2016), reported health-related musculoskeletal outcomes of WB-EMS application (Table 2) in non-athletic cohorts. Eight of these studies considered muscle mass, sarcopenia and sarcopenic obesity as a main study endpoint. One project also addressed (Kemmler et al., 2013; Von Stengel et al., 2015) bone mineral density in postmenopausal women with osteopenia and four other projects gave results on low back pain (LBP) in people with LBP (Grützmacher, 2002; Kemmler et al., 2017c). Another project that determined the effect of WB-EMS on aerobic capacity (Fritzsche et al., 2010) listed lean body mass (LBM) as a subordinated study outcome.

**Table 2:** WB-EMS results on muscle mass and function.

**Authors**	**Attendance, Loss to FU**	**Muscle-/Bone parameter (parameter: % change, EMS vs. CG)**	**SMD d'**	**Physical functioning (parameter, % change, EMS vs. CG)**	**SMD d'**	**Adverse effects intervention**
Fritzsche et al., 2010	n.g., 0%	LBM: 5%; p: n.g.	–	Spiro-Ergometry (bicycle); maximum power (W) at an-aerobic threshold 30%, p: n.g.; VO_2_ threshold: 25%; p: n.g.	–	None
Kemmler et al., 2010b, 2015c	98, 0%	LBM: 0.4%^n.s^ vs. −0.9%^n.s^; *p* = 0.046	0.75	LP (strength): 10%^*^ vs. −5%^n.s^. *p* = 0.001 Trunk exten.: 10%^*^ vs. −6%^n.s^; *p* = 0.006	1.53 1.43	None
Kemmler et al., 2010a, 2015c	78, 7%	LBM: 1.1%^n.s^ vs. −0.6%^n.s^; *p* = 0.032 ASSM: 0.8%^n.s^ vs. −1.1%^n.s^; *p* = 0.024	0.81 1.02	LP (strength): 21%^*^ vs. 5%^n.s^; *p* = 0.001 LP (power): 12%^*^ vs. 3%; *p* = 0.001	1.33 1.13	none
Kemmler et al., 2013, 2014; Von Stengel et al., 2015	79, 16%	LBM: 0.8%^*^ vs. −0.8%^*^; *p* = 0.008 ASSM: 0.4%^n.s^ vs. −1.5%^*^; *p* = 0.009 BMD LS: 0.6 ± 2.5% vs. −0.6 ± 2.4%; *p* = 0.051 BMD hip: −0.9 ± 1.9% vs. −1.0 ± 2.3%; n.s.	0.71 0.71 0.49 0.10	LP (strength): 10%^*^ vs. 0%^n.s^; *p* = 0.003 Trunk exten.: 10%^*^ vs. −2%^n.s^; *p* = 0.001	1.10 0.82	None
Kemmler and Von Stengel, 2013	76, 9%	ASSM: 0.5%^n.s^ vs. −0.8%^*^; *p* = 0.025 Upper Leg LBM: 0.5%^n.s^ vs. −0.9%^*^; *p* = 0.033	0.69 0.65	LP (strength): 9%^*^ vs. 1%^n.s^; *p* = 0.010	0.82	None
Kemmler et al., 2015d, 2016c	*90, 9%*	LBM: 0.9%^*^ vs. HIT: 1.2%^*^; p = 0.395 ASSM: 1.5%^*^ vs. HIT: 1.9%^*^; p = 0.341	*−0.25 −0.28*	LP (strength): 8%^*^ vs. HIT: 14%^*^p = 0.348 Trunk extension.: 12%^*^ vs. HIT: 10%^*^; p = 0.609	*−0.28* *0.15*	*None*
Kemmler et al., 2015c	*94, 8%*	*ASMM: 0.3%^*n*.*s*^; vs. −0.3%^*n*.*s*^; p = 0.166*	*0.58*	LP (strength): 24%^*^ vs. 13%^*^; p = 0.001	*1.67*	*None*
Kemmler et al., 2016d,e	88, 10%	ASMM: 2.5%^*^ vs. −1.2%^n.s^; *p* = 0.001	1.23	LP (strength): 21%^*^ vs. 2% ^n.s^; *p* = 0.002 LP (power): 11%^*^ vs. 3% ^n.s^; *p* = 0.064 Gait speed: 7%^*^ vs. −3%^n.s^; *p* = 0.044 Chair rise Test: −6%^*^ vs. −1%^n.s^; *p* = 0.074	0.86 0.64 0.75 0.63	None
Kemmler et al., 2017a,c	91, 9%	LBM: 3.3%^*^ vs. −0.2%^n.s^; *p* = 0.001 ASSM: 2.5%^*^ vs. −1.1%^n.s^; *p* = 0.001	1.85 1.66	LP (strength): 11%^*^ vs. 2%^n.s^; *p* = 0.001 Gait speed: 3%^*^ vs. −5%^*^; *p* = 0.001 Grip-strength: 6%^*^ vs. −1% ^n.s^; *p* = 0.034	1.20 1.05 0.82	None
Özdal and Bostanci, 2016	*n.g., n.g*.	*LBM: 0.2%^*n*.*s*^ vs. −0.5%^*n*.*s*^; p: n.g*.	*0.12*	*Not assessed*	–	*n.g*.
van Buuren et al., 2013	*n.g., n.g*.	*No data reported*		VO_2_threshold: 30%^*^ vs. local EMS: 18%^*^; p: n.s. VO_2_peak: 23%^*^ vs. local EMS: 9^*^; p: n.s. Max. power (Wmax): 6%^*n*.*s*^ vs. local EMS: 15%^*^; p: n.g.	*0.61* *0.90* *−0.91*	*n.g*.
Vatter, 2003	100, 20%	No data reported		Latissimus pulldowns: 12%^*^ vs. 1%; *p* < 0.001	0.93	None

ASSM, Appendicular skeletal muscle mass; BMD, Bone mineral density; LBM, Lean body mass; LP, leg press; n.g., not given; n.s., non-significant;

* *p < 0.05; SMD, standardized mean difference (Cohens d'); italic: active control group*.

Of importance, the assessment of lean body mass (LBM) or muscle mass is not trivial. Apart from study length, in particular the methodology of assessment is crucial for a valid assessment of LBM, muscle mass, and fat mass. Only six studies addressing body composition used the “gold standard” of dual energy x-ray absorptiometry (DXA, Kemmler et al., 2010a, 2014, 2016c,e) or direct-segmental, multi-frequency Bio-Impedance Analysis (DSM-BIA; Inbody 770, Seoul Korea, Kemmler et al., 2015c, 2017c). The other studies, however, did not specify the testing device used (Fritzsche et al., 2010) or applied outdated methods/technology (caliper method, RMR, Kemmler et al., 2010b, single frequency BIA method, Vatter, 2003; Özdal and Bostanci, 2016).

With one exception (Kemmler et al., 2015c) which reported borderline non-significant effects, all the WB-EMS trials that reliably addressed lean body/muscle mass (Kemmler et al., 2010a, 2014, 2016c,e, 2017c) reported significant increases and/or significant effects (i.e., differences between WB-EMS and control group) on LBM and related parameters. However, one study (Özdal and Bostanci, 2016) showing the limitations described above failed to trigger relevant effects on LBM after WB-EMS with and without voluntary exercises (Table 2).

It is difficult to decide which groups benefit most from WB-EMS application. Although one might expect that frailer cohorts might have the largest potential to adapt, we did not detect any difference between older cohorts with sarcopenic obesity (Kemmler et al., 2016e, 2017c) and men 30–50 years old (Kemmler et al., 2016c).

Although WB-EMS can be thus considered as an option for subjects who do not undertake resistance type exercise anyway, a corresponding comparison of the effectiveness on muscle mass parameters is meaningful. Only one study addressed this issue and directly compared WB-EMS with a “state-of-the-art” high-intensity resistance exercise training (HIT) (Kemmler et al., 2016c) of comparable time effectiveness. This study demonstrated significant increments for both groups with slightly higher (*p* ≥ 0.341) effects on LBM and appendicular skeletal muscle mass (ASMM) in the HIT group (Table 2).

Switching from sarcopenia to osteopenia, only one study (Von Stengel et al., 2015) addressed bone as a study endpoint. After 12 months of WB-EMS application 1.5 × 20 min/week (Table 1) with osteopenic women 70 years+, the authors reported a borderline (non-)significant effect for BMD at the lumbar spine without any relevant effect at the hip region of interest. Considering the high effect of WB-EMS on muscle mass and strength and the close interaction of muscle and bone (Ferretti et al., 2003; Qin et al., 2010) frequently postulated, this result was not expected (Table 2).

With respect to low back pain (LBP), four projects were identified as determining the effect of WB-EMS on back pain in people with relevant complaints. One study, a non-published masters thesis (Grützmacher, 2002), reported in summary a reduction of dorsal pain frequency in 89% of their 49 WB-EMS applicants. Of importance, the highest improvements were given for the lumbar spine region. Two participants reported negative changes of LBP after WB-EMS, however. Unfortunately, the study did not implement a non-training CG and did not adequately report the training effects, thus the evidence of this study remains limited. The same is true for the study by Fritzsche et al. (2010), which reported that back pain in participants with corresponding complaints “was completely eliminated after a few WB-EMS sessions.” More evidence-based, another masters thesis (Vatter, 2003) and NCT reported significant positive WB-EMS effects in the subgroup with back complaints (*n* = 117). Briefly, the author reported significant WB-EMS-induced improvements of frequency and intensity of back pain versus no changes in the CG, with the highest positive effects observed for the lumbar spine region. Finally, a recent meta-analysis of individual patient data (Kemmler et al., 2017b), derived from five RCTs that applied similar WB-EMS protocols in untrained subjects >60 years with chronic, unspecific LBP, confirmed the favorable effect of WB-EMS on LBP with adequate evidence. Overall, the authors reported significant positive changes after WB-EMS (*p* < 0.05) and significant effects for low back pain intensity (*p* = 0.008) and frequency (*p* = 0.035) compared with non-training controls.

### Conclusion: musculoskeletal risk factors and diseases

In summary, WB-EMS is particularly effective for addressing muscle mass in untrained people independently of their age, sex and muscle status (i.e., healthy vs. sarcopene). Further, there is considerable evidence for a significant favorable effect of WB-EMS on low back pain, while the corresponding effect on bone strength (i.e., BMD) was only borderline effective and should be reevaluated.

### (2) WB-EMS-induced changes of muscle strength, functional capacity and related parameters

The positive effect of therapeutic, locally applied EMS on muscle weakness in athletes (Filipovic et al., 2012), healthy subjects (Filipovic et al., 2011), or people with chronic diseases (Jones et al., 2016) is well recognized. Apart from the volume of stimulated muscle groups, mechanisms and the mode of action of WB-EMS does not differ relevantly from locally applied EMS. Thus, in general it is reasonable to transfer the results of locally applied EMS interventions (Filipovic et al., 2011, 2012; Jones et al., 2016) to the topic of WB-EMS, at least under the premise of comparable stimulation protocols. This estimation was confirmed when roughly comparing the results for quadriceps/leg extensor strength changes after local EMS (Jones et al., 2016) vs. WB-EMS (see below) protocols in untrained older cohorts. In detail, there is some evidence that locally applied EMS was slightly more favorable for increasing strength-related outcomes compared with WB-EMS (Filipovic et al., 2012)—at least in athletes. Speculatively, this may be due to the optimal placement of a single electrode and concentration on a single region when exercising with high voluntary intensity.

Eleven WB-EMS studies reported strength and/or functional parameters as a primary or secondary study endpoint. In summary, all the studies that determined muscle strength, power and/or functional parameters (e.g., gait speed, handgrip strength) (Table 2) reported significant improvements that differ significantly from their corresponding non-, or semi-active control groups. Further, most of the trials with an active CG stated significantly (Kemmler et al., 2010b, 2015c) more favorable effects in the subgroups that applied WB-EMS and exercise compared with the exercise-only subgroups. Quantifying this net effect for leg and hip extensor strength (leg press), WB-EMS effects averaged 8–19% (ES: d': 0.82–1.53), when compared with a semi-active or inactive CG and−6% to 11% (ES: d': −0.28 to 1.67) when compared with an active CG (Table 2). When addressing the relevance of adjuvant slight voluntary movements during WB-EMS-application, one RCT provided (Kemmler et al., 2015c) further information. This study compared the effect of two protocols conducted once a week for 20 min applied in a supine position, however with and without slight movements during identical WB-EMS settings. The authors (Kemmler et al., 2015c) reported significant leg and hip extensor (leg press) strength enhancements in both groups, the WB-EMS application with the slight movements without any resistance significantly outperformed the “WB-EMS only” approach (24 vs. 13%), however.

Studies that compared the effect of WB-EMS protocols vs. conventional resistance type exercise are rare. Only one study (Kemmler et al., 2015d, 2016c) directly compared the effect of WB-EMS versus a comparably time-effective HIT protocol (Gießing, 2008). Of importance, the authors reported significant increases for both exercise protocols. In detail, HIT was slightly more favorable in affecting leg and hip extension strength (leg press), while WB-EMS was non-significantly more effective in improving back extension strength (Table 2).

Functional tests with older people were conducted by three projects (Von Stengel et al., 2015; Kemmler et al., 2016d,e, 2017a,c). All the projects predominately focused on the effect of WB-EMS on body composition and functional parameters in woman (Kemmler et al., 2014, 2016d,e; Von Stengel et al., 2015) and men (Kemmler et al., 2017a,c) 70 years and older with sarcopenic obesity. Significant positive effects on habitual gait velocity (7–10%) and grip strength (5–10%) were reported in all of them. However, although one study reported significant positive changes in a counter-movement jump and chair rising test, the study just failed (*p* = 0.064 and 0.074) to determine significant effect (i.e., differences WB-EMS vs. CG).

With respect to endurance-related parameters two projects with three studies (Fritzsche et al., 2010; van Buuren et al., 2013, 2014) determined WB-induced effects in non-athletic cohorts. Firstly, Fritzsche et al. (2010) reported a significant improvement in power output during cycle-ergometry (31%) combined with a 25% increase (*p* < 0.05) in VO_2_max for patients with chronic heart failure (Table 2). In his combined analysis of men and women (van Buuren et al., 2013), and in his sub-analysis of men (van Buuren et al., 2014) with CHF, van Buuren et al. reported significant changes of VO_2_peak and VO_2_ at the anaerobic threshold in the WB- and local EMS-group. Maximum power output (Watt_max_) and power output at the anaerobic threshold (W_threshold_) improved in both sub-groups, however significance was realized only for W_threshold_ in the local EMS-group. Unfortunately, only the study with men (van Buuren et al., 2014) listed group differences, which were not significant for changes in gas exchange parameters (Watt_max_ not given), however.

### Conclusion: muscle strength, functional capacity, and related parameters

In summary, the WB-EMS-induced effect on muscle strength and—to a slightly lesser degree—on physical functioning can be considered significant and clinically relevant, independently of the cohort addressed. One study (Kemmler et al., 2015c) illustrated the superiority of adjuvant slight exercises/movements during the impulse phase vs. passive WB-EMS application on maximum strength. Considering more complex abilities (i.e., dynamic and static balance) and corresponding risk factors (i.e., falls), movement/exercises applied during WB-EMS should be more customized (e.g., by perturbation). This might indicate the need to reduce impulse intensity to a level that enables the proper execution of the dedicated exercise, a strategy predominately applied during WB-EMS application with athletes (e.g., Wahl et al., 2014; Filipovic et al., 2015; Wirtz et al., 2016).

The effect of WB-EMS on endurance performance parameters is less distinct. Significant changes of VO_2_max as determined by two projects (Fritzsche et al., 2010; van Buuren et al., 2013, 2014) were not consistently accompanied by significant improvements of valid endurance performance parameters (i.e., Watt_max_, Watt_threshold_). Further, the eligible studies listed in this review (Fritzsche et al., 2010; van Buuren et al., 2013, 2014) focused on very inefficient patients with CHF, i.e., a cohort with very low adaptive thresholds. Studies with superimposed WB-EMS during endurance exercise in athletic populations suggest, however, that EMS application could be beneficial for aerobic performance enhancements in athletes and in patients who cannot perform high workloads (Wahl et al., 2012).

### (3) WB-EMS-induced changes of cardio-metabolic risk factors and diseases

When considering body fat as a cardio-metabolic risk factor and obesity as a cardio-metabolic disease, eleven projects (Vatter, 2003; Fritzsche et al., 2010; Kemmler et al., 2010a,b, 2014, 2016b,c,e, 2017c; van Buuren et al., 2013; Özdal and Bostanci, 2016; Wittmann et al., 2016) focused on - or at least reported data on—this issue.

Two projects (Fritzsche et al., 2010; van Buuren et al., 2013, 2014) focused on the effect of WB-EMS in patients with chronic heart failure (CHF). While Fritzsche et al. (2010) focused on secondary prevention, and van Buuren et al. (2013) reported a significant raise of left ventricular ejection fraction (LVEF) of 13% after WB-EMS versus a slight increase of 6% (*p* = 0.27) in the local EMS-group. In a sub-study of this research group (van Buuren et al., 2013) that focused on men only, van Buuren et al., 2014 provided comparable results (LVEF: WB-EMS: 15%, *p* < 0.05 vs. EMS: 7%^n.s.^). Unfortunately, the authors did not provide significance levels of the corresponding group differences (Table 3).

**Table 3:** WB-EMS results on cardio-metabolic parameters.

**Authors**	**Atd, LtF**	**Overall cardiac/metabolic effects changes EMS vs. CG, p**	**Fat mass % change, EMS vs. CG, p**	**SMD d'**	**Other cardiometabolic risk factors % change, EMS vs. CG, p**	**Adverse effects intervention**
Fritzsche et al., 2010	n.g., 0%	Significant increase of VO_2_max and power out-put in the WB-EMS group (no CG implemented)	TBF: −2 to 3%; p: n.g.	–	MAP: −3.6%^*^ (no CG)	None
Kemmler et al., 2010b, 2015c	98, 0%	No data reported	TBF: −8.6%^*^ vs. +1.4%^n.s^; *p* = 0.001 WC: −2.3%^*^ vs. +1.0%^n.s^; *p* = 0.001	1.37 1.64	No data reported	None
Kemmler et al., 2010a, 2015c	78, 7%	No data reported	TBF: −6.3%^*^ vs. −1.4%^n.s.^, *p* = 0.008 ABF: −6.8%^*^ vs. −0.9%^n.s.^, *p* = 0.004 WC: −5.7^*^ vs. – 3.2%^*^; *p* = 0.023	1.23 1.33 1.10	No data reported	None
Kemmler et al., 2014	79, 16%	No data reported	TBF: −0.8%^n.s^ vs. −0.4%^n.s^; *p* = 0.87 ABF-0.4%^*^ vs. 0.2%^n.s^; *p* = 0.069 WC: −1.3^*^ vs. 0.6%^n.s^; *p* = 0.006	0.05 0.47 0.74	No data reported	None
Kemmler and Von Stengel, 2013	76, 9%	No data reported	ABF-1.2%^*^ vs. 2.4%^n.s^; *p* = 0.038 WC: −1.1^*^ vs. 1.0%^n.s^; *p* = 0.007 ULFat: −0.8^n.s^ vs. 1.0%^n.s^; *p* = 0.050	0.63 0.85 0.60	No data reported	None
Kemmler et al., 2015d, 2016b	*90, 9%*	MetS-Z: EMS −1.16^*^ vs. HIT: −0.51^*^; p = 0.096	TBF: −4.4%^*^ vs. HIT: −3.7%^*^; p = 0.829 ABF: −5.9%^*^ vs. HIT: −5.2%^*^, p = 0.499 WC: −3.4%^*^ vs. HIT: −2.1%^*^; p = 0.306	*0.07* *0.02* *0.30*	MAP: −4.9%^*^ vs. HIT: −3.6%^*^; p = 0.306 HDL-C/TC: −2.2%^*n*.*s*^ vs. −2.7%^*n*.*s*^; p = 0.93 Glucose: −4.5%^*^vs. 1.8%^*n*.*s*^; p = 0.025 TG: 5.9%^*n*.*s*^ vs. −7.2%^*n*.*s*^; p = 0.200	*None*
Kemmler et al., 2015c	*94, 8%*	*No data reported*	TBF: −2.5%^*^ vs. −1.1%^*n*.*s*.^; p = 0.052	*0.56*	*No data reported*	*None*
Kemmler et al., 2017a,c	91, 9%	No data reported	TBF: −6.6%^*^ vs. 1.0%^n.s^; *p* = 0.001	1.07	No data reported	None
Özdal and Bostanci, 2016	*n.g., n.g*.	*No data reported*	TBF: −15.1%^*^ vs. −1.0%^*n*.*s*^; p: n.g. Trunk fat: 20.3% vs. −0.3%^*^; p: n.g.	*2.66* *4.66*	*No data reported*	*n.g*.
van Buuren et al., 2013, 2014	*n.g*.	LVEF: 13%^*^ vs. local EMS: 6%^*n*.*s*^; p: n.g. Men only: LVEF: 15%^*^ vs. EMS: 7%^*n*.*s*^; p: n.g. Significant increase of VO_2_max (see Table 2)	*TBF: 0.8%^*n*.*s*^; vs. −3.0%^*n*.*s*^; p: n.g*.	*−0.60*	*Syst. RR: 0%^*n*.*s*^ vs. −3%^*n*.*s*^; p: n.g. Diast. RR: 0%^*n*.*s*^ vs. −3%^*n*.*s*^; p: n.g*.	*n.g*.
Vatter, 2003	100, 20%	No data reported	TBF:−1.4%^*^ vs. 6.7%^n.s^.; p < 0.05	0.88	No data reported	None
Kemmler et al., 2016e; Wittmann et al., 2016	88, 10%	MetS-Z: −0.36^n.s^ vs. 0.26^n.s^; p: n.s. ProteinandWB-EMS: −0.84^*^ vs. 0.26^n.s^; *p* = 0.012	TBF: −0.9%^*^ vs.−0.8%^n.s^; *p* = 0.746 WC: −1.5%^*^ vs. 0%^n.s^; *p* = 0.036	0.05 0.66	MAP: −8.1%^*^ vs. −2.1%^n.s^; *p* = 0.04 HDL-C: −1.8%^n.s^ vs. −3.5%^*^; n.s. Glucose: −3.1%^n.s^ vs. −3.6%^n.s^; n.s. TG: 2.2%^n.s^ vs. 8.0%^n.s^; n.s.	None

*ABF: Abdominal body fat; Atd: Attendance rate; LtF: Lost to follow-up rate; MetS-Z: Metabolic syndrome Z-score; MAP: mean arterial pressure; TBF: Total body fat; TG: Triglycerides; ULF: Upper leg fat mass; WC: Waist circumference; italic: Active control*.

* p < 0.05

Two studies reported the effect of WB-EMS on the Metabolic Syndrome (MetS) in women 70+ with sarcopenic obesity (Wittmann et al., 2016) or predominately overweight-obese untrained men 30-50 years old (Kemmler et al., 2016b). The latter study (Kemmler et al., 2016b) presented a significant improvement of the MetS Z-Score after 16 weeks of low-frequency WB-EMS that was considerably higher (*p* = 0.096) compared with the high intensity resistance training (HIT) control group. Results of the study of Wittmann et al. (2016) were less distinct. While isolated WB-EMS did not induce a significant improvement of the MetS-Z Score, a combination of low dose protein supplementation (0.33 g/d/kg body mass) and WB-EMS demonstrate more favorable, significant results (Table 3). Looking behind the covariates of the MetS, waist circumference and mean arterial pressure (MAP) demonstrated the most pronounced reduction. The latter result was confirmed by Fritzsche et al. (2010) and rejected by van Buuren et al. (2013, 2014), who reported either significant reductions or slight increases of systolic and diastolic RR after WB-EMS in patients with CHF (Table 3).

No relevant changes or effects were observed for HDL-C, triglycerides or fasting glucose that further constitute the MetS criteria (Expert-Panel, 2001; Alberti et al., 2006); however, there is some evidence that combined WB-EMS and protein-supplementation protocols might be more effective for generating a corresponding effect (Table 3).

With respect to body fat or obesity, the present data (Table 3) suggested that the WB-EMS-induced positive effect on total and abdominal body fat parameters is even higher than the corresponding effect on muscle mass. Indeed, independently of the testing method, all the studies (Vatter, 2003; Fritzsche et al., 2010; Kemmler et al., 2010a,b, 2014, 2016c,e, 2017c; Özdal and Bostanci, 2016; Wittmann et al., 2016) listed significant or at least favorable WB-induced effects on total and/or abdominal body fat. Only one study (Özdal and Bostanci, 2016) provided confusing results, with very high total (−15.1 ± 6.2% vs. CG: −1.0 ± 4.2) and abdominal (−20.3 ± 6.2% vs. CG: 0.3 ± 0.8%) fat reductions in the active (i.e., with adjuvant exercises of moderate intensity) WB-EMS group, while “passive” (non-voluntary) WB-EMS did not relevantly affect body fat parameters. This result would imply that favorable effects on body fat could be attributed exclusively to the voluntary exercise without any relevant contribution of WB-EMS. Although changes of body fat parameters may be indeed higher when applying adjuvant movements (Kemmler et al., 2015c), the dimension of the effects on abdominal and total body fat effects remained debatable at least when considering the short training period in these normal weighted females without any nutritional intervention (Table 3).

### Conclusion: cardio-metabolic risk factors and diseases

The effect of WB-EMS on cardiac and metabolic risk factors is quite complex and may be triggered by the clinically relevant reduction of total and abdominal body fat reported by most studies (Table 3). Indeed, central obesity, embracing subcutaneous and intraabdominal adipose tissue, provokes low grade inflammation related to a plethora of negative affects including increased insulin resistance (Zhang et al., 2015), atherosclerosis (King and Ajjan, 2017), hypertension (Lakoski et al., 2011), dyslipidemia (Bastien et al., 2014), and reduced left ventricular function (Gaborit et al., 2012). In parallel to our results, existing data for resistance exercise, the exercise type most akin to WB-EMS, confirmed the favorable effect of RT-type exercise on obesity, and related cardio-metabolic diseases (Strasser and Schobersberger, 2011; Strasser et al., 2012; Strasser and Pesta, 2013).

### (4) risk factors of WB-EMS application and adverse effects during WB-EMS interventions

Due to the high volume of simultaneously stimulated muscle area (up to 2,800 cm^2^) and the possibility to exercise with a supramaximal intensity that is able to generate complete tetanus of the muscle, WB-EMS is a perfect candidate (Koch et al., 2014) for inducing severe muscle damage and exertional rhabdomyolysis.

As mentioned, we addressed this issue with an independent literature research for the topic “risk factors of WB-EMS application,” while the issue “adverse effects during interventions” was already addressed by the aforementioned literature research strategy on WB-EMS interventions. Briefly, a comprehensive search of electronic databases was conducted through PubMed, Scopus, Cochrane, Web of Science, PsycINFO and Eric for all articles published in English and German up to October 31, 2017 on the effect of WB-EMS-induced risk factors. The literature search was constructed around search terms for “Whole-Body Electromyostimulation and risk factors” and “Whole-Body Electromyostimulation and rhabdomyolysis.” A standard protocol for this search was developed and controlled vocabulary (Mesh term for MEDLINE) was used. Key words and their synonymous were used to sensitize the search by using the following query: “Whole-Body Electromyostimulation” or “whole body electrostimulation” or “whole body myostimulation” AND “risk factors” or “rhabdomyolysis” or “creatine kinase” or myoglobin” or “acute renal failure” or “renal damage” or “cardiac arrest” or “electrolytes” AND “adults.” Corresponding German key words were used. Further, reference lists of the included studies were searched manually. Duplicate publications were identified by comparing author names, treatment comparisons, publication dates, sample sizes, and outcomes. Randomized controlled trials (RCT), non-randomized controlled trials (NCT), meta-analysis of individual patient data, peer reviewed scientific thesis, and case control studies and case series were included in the review. Data extraction was conducted by SvS and WK following the process provided above.

All the articles that satisfied the predefined inclusion criteria (Figure 1) were independently assessed for risk of bias by two independent raters (WK and SvS) using the PEDro scale (Sherrington et al., 2000; de Morton, 2009) introduced above, or the quality assessment checklist for case series provided by Moga et al. (2012). In a nutshell, this checklist is based on a modified Delphi technique (Jones and Hunter, 1995) and allows a maximum of 18 score points. Discrepancies in the quality assessment between the raters were discussed with a third assessor until a consensus was reached.

In summary, six projects that reported low-frequency WB-EMS-induced medical risk factors were identified. Five studies were case series (Kästner et al., 2014; Finsterer and Stollberger, 2015; Hong et al., 2016; Malnick et al., 2016; Herzog et al., 2017) with one or two subjects, another project included two RCTs (Kemmler et al., 2015b; Teschler et al., 2016) with 26 participants. Applying the PEDro scale to the latter study we (SvS, WK) allocated seven score points. With respect to the three case series, score points of between 9 and 11 (of 18 score points) were allocated. Briefly, the low number of participants in the study I particular prevents a higher quality rating.

The cohorts of these eligible studies vary from physically highly (Kästner et al., 2014; Kemmler et al., 2015b; Teschler et al., 2016) to moderately trained (Finsterer and Stollberger, 2015; Kemmler et al., 2015b; Teschler et al., 2016) and untrained (Hong et al., 2016) young to middle-aged men and women (Table 4). None of the participants have experience in WB-EMS training and all the studies reported the WB-EMS-induced rhabdomyolysis during the initial session(s). Although not adequately addressed in two articles (Finsterer and Stollberger, 2015; Hong et al., 2016), it is obvious that the studies used supervised standard WB-EMS protocols (bipolar, rectangular, 85 Hz, 350 μs, intermitted 4 s-4 s). Unfortunately, only one study (Kemmler et al., 2015b; Teschler et al., 2016) reported the relative intensity of the WB-EMS application (Table 4).

**Table 4:** Characteristics of WB-EMS-studies that reported or induced rhabdomyolysis in various cohorts.

**Author**	**Study- Design**	**Sample size (*n*)**	**Exercise Status**	**WB-EMS Status**	**Sex, Age (MV ± SD)**	**Intervention**	**Result**
Hong et al., 2016	Case report	1	Healthy, untrained	Untrained, second session	W, 37 years.	60 knee push-ups in 20 min; supervised, WB-EMS protocol: n.g. (presumably a standard protocol Table 1)	CK: 5.400 IU/I; myoglobin: 264 ng/ml Urinary parameters in the normal range, no renal involvement
Finsterer and Stollberger, 2015	Case report	1	Metabolic myopathy, trained	Untrained, initial session	W, 32 years.	15 min, supervised; WB-EMS protocol: n.g. (presumably a standard protocol Table 1)	CK: 86,000 IU/l; Myoglobin: 1,930 μg/l; cola-colored urine, normal glomerular filtration rate (GFR)
Herzog et al., 2017	Case report	2	Healthy	Untrained (?), initial session	M, 32, 32 years.	n.g., (presumably a standard protocol, Table 1)	CK: 71,300 and 24,500 IU/l, urinary parameters in the normal range, no renal or hepatic involvement
Kästner et al., 2014	Case report	2	Healthy, Athletes	Not given	M, 17, 19 years.	20 min, supervised; WB-EMS protocol: n.g. (presumably a standard protocol Table 1),	CK: 30,000 and 240,000 IU/l; myoglobin 6,800 μg/l, cola- or normal colored urine, normal GFR
Malnick et al., 2016	Case report	1	Not given	Not given	M, 20 years.	n.g. (gym based whole body EMS exercise supervised by a fitness professional).	CK: 129,000 IU/l, no renal involvement^a^
Kemmler et al., 2015b; Teschler et al., 2016	Clinical study	26	Healthy, Moderate—highly trained	Untrained, initial session	M + W, 32 ± 8 years.	1–2 sets, 10 exercises, 6–8 reps in a standing position; **bipolar, rectangular, 350** μ**s**; 20 min with **85 Hz**. 4 s impulse−4 s rest, 8 (very hard+ to extremely hard) on Borg CR-10	Peak-CK: 29,000 ± 34,000 IU/l; maximum individual CK: 144,000 IU/l; Peak-myoglobin: 2,800 ± 2,200 μg/l; no relevant changes of electrolytes, urinary parameters in the normal range; no cola-colored urine, normal GFR.

*CK, creatine kinase; GFR, glomerular filtration rate*.

a *Values reported in the article of Stöllberger and Finsterer (2018)*.

All the studies listed in table 4 reported WB-EMS-induced CK-levels in the range of moderate^1^ to severe^2^ rhabdomyolysis (Visweswaran and Guntupalli, 1999). Myoglobin concentration was also significantly increased, but the effect was less pronounced compared with CK. Cola-colored urine was reported in one study, but indicators of renal damage or failure were not observed. In parallel, no cardiac risk factors caused by rhabdomyolysis-induced changes of electrolytes were demonstrated. Using a pre-specified WB-EMS protocol that mimics commercial standard protocols (Table 1), albeit applied with an inappropriately high intensity (RPE 8-9 on a BORG CR 10 scale), Teschler et al. (Kemmler et al., 2015b; Teschler et al., 2016) focused on the issue of WB-EMS-induced rhabdomyolysis and corresponding health hazards (Table 4). The authors applied supervised WB-EMS with borderline high current intensity to healthy adults who had not conducted WB-EMS training for at least 9 months. Based on normal values, CK and myoglobin increased 117- and 40-fold (Table 4), with peak concentration on day 3–4. Of interest, changes were not related to sex, age, body composition or training status. Although high CK and myoglobin levels did not result in clinical consequences, the corresponding risk may be much more prevalent in less healthy and inadequately prepared people. Addressing this problem of inappropriately high intensity during initial WB-EMS training session(s), the authors (Kemmler et al., 2015b; Teschler et al., 2016) looked at the corresponding long-term effect of WB-EMS. After 10 sessions (1 × 20 session per week) applying a standard WB-EMS program (Table 1) and a final WB-EMS application again with borderline current intensity, the authors observed a very pronounced repeated bout effect (Nosaka and Clarkson, 1995). In detail, WB-EMS-induced changes of CK was significantly reduced (CK-peak: 906 ± 500; <4-fold increase from baseline) and fell in the lower range of dynamic resistance exercise (Koch et al., 2014). Studies that routinely monitor CK values during their WB-EMS intervention (e.g., Fritzsche et al., 2010; Filipovic et al., 2016) confirmed this result.

Thus, the trigger for EMS-induced rhabdomyolysis obviously seemed to be inappropriately (excessively) high current intensity during the initial training sessions. A recent guideline for the safe and effective application of WB-EMS in commercial settings (Kemmler et al., 2016a) tackles this issue and strongly contraindicates high load intensities during the initial sessions and consistent work to failure after the corresponding WB-EMS conditioning period of 10 weeks.

Apart from possible rhabdomyolysis-induced effects on electrolytes with possible impact on the cardiac system, albeit not confirmed by the present studies (Kemmler et al., 2015b; Teschler et al., 2015, 2016), a direct-current-induced effect on cardiac electrical conduction might be a relevant risk of WB-EMS. Consequently, cardiac arrhythmia and pacemakers were considered to be contra-indicated for the application of WB-EMS and listed as such in several distributor manuals. However, the scientific evidence that the current specifications of low-frequency WB-EMS *per se* affect cardiac electrical conduction is not convincing. Apart from chronic heart diseases related to electrical conduction (e.g., cardiac arrhythmia), some studies explicitly applied EMS (review in Adams, 2017 or WB-EMS Fritzsche et al., 2010; van Buuren et al., 2013, 2014) in patients with chronic low heart failure without any negative side effects.

Other undesired metabolic side effects of WB-EMS might be post-exercise weakness, dizziness or headache. Although it is difficult to retrospectively analyze the specific reasons, there is some evidence to link these ailments (1) to an inappropriately high total intensity of the WB-EMS application and/or (2) to poor preparation and post-processing of the WB-EMS session by the user. This includes adequate energy and liquid supply prior to and after the WB-EMS session and abstaining from alcohol and drugs prior to the WB-EMS session. In this context, adequate energy and fluid supply is particularly relevant due to the peak in moderate-high energy demand of a standard WB-EMS session (Kemmler et al., 2012b). Recent recommendations for proper preparation of a WB-EMS session by instructors and users are provided in the WB-EMS guideline (Kemmler et al., 2016a) which is mandatory within the aforementioned licensing procedure for commercial WB-EMS facilities.

With respect to adverse effects, none of the studies listed in Tables 2, 3 reported any adverse or unintended side effect of WB-EMS applications, even though study periods reached up to 12 months of intervention. This is even more notable, since the majority of these studies predominately focused on older, functionally limited and/or morbid cohorts. However, in four projects (Kemmler et al., 2014, 2016c,e, 2017c), one participant in each of the studies quit due to “discomfort during WB-EMS application.”

### Conclusion: risk factors and adverse effects of WB-EMS application and interventions

Briefly, properly applied and guided WB-EMS application according to the present guidelines is a safe training technology predominately, but not only, for unfit and physically limited subjects with low time budgets. However, more properly designed long-term studies have to confirm this result.

### Study limitations

The aim of this systematic review was to provide evidence for the benefits, limitations and risks of low-frequency WB-EMS application on the health of and disease among non-athletic, predominately older cohorts with considerable musculoskeletal, functional and/or cardio-metabolic limitations. Actually, from the qualitative description of the data, most approaches implemented a meta-analysis in order to increase statistical power, address the reproducibility aspect, generate quantitative data and aimed to generate recommendation for effective protocols (Cohn and Becker, 2003). However, as discussed in length elsewhere (Borenstein et al., 2009) it does *not always* make sense to perform a meta-analysis. Present biometric procedures used in meta-analytic research allow the addressing of common limitations of meta-analysis e.g., publication bias (Joober et al., 2012), small study effect (Schwarzer et al., 2015), or quality of included studies (de Morton, 2009). However, the most important issue for interpreting the meta-analysis may be the threshold up to which a study can still be meaningfully included (Greco et al., 2013). With respect to this contribution, the heterogeneity of the manipulation of exercise parameters along with the implementation of active control groups are the main barriers to meaningful meta-analysis and quantifying summary of the few projects that addresses the topic of interest.

Summing up the pitfalls that may aggravate a proper interpretation of the listed results, some limitations should be considered.

1. Due to the fact that WB-EMS is a rather new exercise technology, we decided to include eligible scientific theses in our search strategy. The rational for this approach was that we considered peer-reviewed theses as the first step within scientific research. Unfortunately, we are unable to include scientific theses in our database search, hence the all corresponding projects (i.e., Grützmacher, 2002; Vatter, 2003) were detected by reviewing other sources. This however implies that we might have failed to identify all the relevant theses in this area.
2. We do not focus on *all* health-related parameters evaluated by WB-EMS trials. This relates to the topic of (1) urine incontinence (Schäffer, 2002; Vatter, 2003) and (2) quality of life (QoL) (e.g., Vatter, 2003; Fritzsche et al., 2010; van Buuren et al., 2014). With respect to the first topic, local EMS is a recognized therapy (Jerez-Roig et al., 2013; Schreiner et al., 2013), the additional benefit of the less specific but more elaborate WB-EMS is debatable.
3. Most of the projects included in these systematic reviews implemented an “active” control group. The types of exercise applied in these cohorts vary from “wellness,” “whole body vibration,” “locally applied EMS,” “WB-EMS without exercises” to “state-of-the-art HIT-RT” (Table 1). Thus, changes among the CGs differ from negligible (Kemmler and Von Stengel, 2013) to highly significant (Kemmler et al., 2016c). Consequently, the “effect,” as defined as the difference of intragroup changes in the WB-EMS vs. the CG, might be less prominent in some projects, due to the effectiveness of both protocols.
4. From a methodological point of view, study length or/and assessment methods/tools are critical for a reliable evaluation of more sophisticated parameters. Concerning hypertrophy, only six studies addressing body composition applied the “gold standard” of dual energy x-ray absorptiometry (DXA; Kemmler et al., 2010a, 2014, 2016c,e), or direct-segmental, multi-frequency Bio-Impedance Analysis (DSM-BIA; Inbody 770, Seoul Korea: Kemmler et al., 2015c, 2017c), that have been proven to be comparably reliable (Ling et al., 2011; Von Stengel et al., 2013). The other studies, however, did not specify the BIA technique used (Fritzsche et al., 2010) or applied outdated methods/technology (caliper methods, Resting Metabolic Rate, Kemmler et al., 2010b, single frequency BIA method, Özdal and Bostanci, 2016). Additionally, studies shorter than 10 weeks (e.g., Vatter, 2003; Özdal and Bostanci, 2016) may be unable to determine relevant LBM changes, at least when applying less reliable testing methods.
5. This limitation of short intervention periods may also affect the regulation of exercise intensity and the corresponding adaptation. Bearing in mind that the high muscular and metabolic stress of the initially unfamiliar WB-EMS condition (Teschler et al., 2016, 2018) led to a brief conditioning period being suggested (Kemmler et al., 2016a), the time frame of some projects might be too short to generate effective WB-EMS effects.
6. As with conventional exercise, the application of an above-threshold strain intensity is a key factor for effective WB-EMS protocols. However, the proper regulation of stimulation intensity is a rather challenging problem. In the absence of objective parameters that reliably and comprehensively graduate the intensity of stimulation, most WB-EMS projects (Table 1) applied RPE-based methods adjusted for each stimulated region. However, even assuming that all of the study participants complied with the RPE prescribed, we are unable to verify whether the prescribed exercise intensity is adequate for affecting the given outcome. Unfortunately, no recent WB-EMS study has reliably focused on the dose/response or minimum effective strain intensity issue of WB-EMS application. Thus, a main limitation might be that WB-EMS projects that failed to produce a given outcome might have applied insufficient exercise intensities at least when additionally scheduling a low exercise frequency.
7. We failed to list this systematic review in PROSPERO because we were beyond the point of completing data extraction when we submitted the data for registration. However, since we intended a quantitative analysis we had to check the corresponding studies initially.

## General conclusion

There is considerable evidence that “low-frequency WB-EMS” /i.e., <100 Hz significantly and clinical relevantly increases health-related parameters in moderately trained and untrained middle-aged to older non-athletic cohorts. The most prominent effect of WB-EMS is, however, the significant impact on body composition. In this context, sarcopenia and sarcopenic obesity in the elderly might be the most promising targets; however, functional aspects and to a lesser extent cardio-metabolic parameters can also be favorably addressed by WB-EMS. With respect to the dimensions of WB-EMS-induced effects, one study with untrained middle aged men (Kemmler et al., 2016c) reported that the effect on body composition (i.e., muscle- and fat mass), muscle strength and cardio-metabolic outcomes were similar to a less time effective and more challenging high intensity resistance exercise training with a work to failure+ protocol. Regarding their applicability, a recent study successfully applied a “high effort training” protocol (Steele et al., 2017b) similar to HIT in people 61-80 years old. Nonetheless, the acceptance of a HIT protocol by sedentary older people remains limited; thus, HIT may not be a genuine exercise training option for most older people. Concerning the corresponding attractiveness of WB-EMS, all the projects reported high adherence with low drop-out (0–10%) and high attendance (76–100%) rates. However, one main reason for the attractiveness might not relate to the WB-EMS technology *per se*, but to the high level of assistance, supervision and interaction between instructor and the maximum of two participants: in effect, a kind of personal training. Of further crucial importance, none of the studies, even when focusing on the vulnerable cohort functionally limited and/or morbid older people (Fritzsche et al., 2010; Kemmler et al., 2010a,b, 2014, 2015c, 2016c,e, 2017c), reported any adverse or unintended side effect of WB-EMS application, even though study periods ran for up to 12 months of intervention. Thus, low-frequency WB-EMS can be considered as a safe training technology at least when practiced in the closely supervised setting applied by all the projects listed above. From the studies included, we think it is legitimate to generalize the results of this review at least on the cohorts of sedentary middle aged to older people unable or unmotivated to join conventional exercise programs. For these people WB-EMS might be a reasonable option for improving health-related outcomes including body composition and physical fitness.

One may rightly argue that WB-EMS did not achieve the enormous comprehensive potential of “exercise” on health-related parameters in middle-aged and older adults (e.g., Macaluso and De Vito, 2004; ExtraMatch-Collaborative, 2006; Marques et al., 2011; Peterson et al., 2011; Ismail et al., 2012; Pattyn et al., 2013; Yang et al., 2014; Searle et al., 2015; Inder et al., 2016; Straight et al., 2016; Chieffi et al., 2017a,b; Sherrington et al., 2017). However, similar to all other types of exercise, WB-EMS did not affect all aspects of physical performance and health, thus ideally it would have to be combined with endurance, flexibility and relaxation exercises that were not addressed in conventional WB-EMS application.

Summing up the topic of health-related aspects of WB-EMS application, one has to bear in mind that evidence-based research seriously addressing this rather novel exercise-training technology is at an early stage. Correspondingly, present studies focus on the most promising effects of WB-EMS, i.e., musculoskeletal, cardiometabolic and functional outcomes.

Consequently, there is a need for more high-end RCTs generated by other independent research groups that clearly confirmed the present results predominately published by our own working group and further address more complex health related parameters e.g. neuronal and cognitive risk factor and diseases. Additionally, studies that focus on specified WB-EMS protocols for dedicated research issues should be implemented in the near future.

## Author contributions

SvS, MS, AW, SW, and WK designed the study, completed data analysis and/or interpretation and drafted the manuscript. AF, HK, JB, and MF contributed to study conception and design and revised the manuscript. WK accepts responsibility for the integrity of the data sampling, analysis and interpretation.

### Conflict of interest statement

The authors declare that the research was conducted in the absence of any commercial or financial relationships that could be construed as a potential conflict of interest.

## Footnotes

^1^11 to 50-fold increase of the “normal” concentration (i.e., 40–170 U/l).

^2^>50-fold increase of the “normal” concentration (i.e., 40–170 U/l).
